# Tensile Strength Analysis of Thin-Walled Polymer Glass Fiber Reinforced Samples Manufactured by 3D Printing Technology

**DOI:** 10.3390/polym12122783

**Published:** 2020-11-25

**Authors:** Jerzy Bochnia, Malgorzata Blasiak, Tomasz Kozior

**Affiliations:** Department of Manufacturing Technology and Metrology, Kielce University of Technology, 25-314 Kielce, Poland; jbochnia@tu.kielce.pl (J.B.); mdrab@tu.kielce.pl (M.B.)

**Keywords:** 3D printing, glass fibers, polymers, PA 3200 GF, tensile strength

## Abstract

The paper describes the mechanical properties, determined on the basis of a tensile strength test of a composite material based on glass-fiber reinforced polyamide and obtained by Selective Laser Sintering—SLS. The material used is PA 3200 GF. Thin walled samples with non-standard nominal thicknesses of 1, 1.4 and 1.8 mm, manufactured in three printing directions X, Y and Z, were used. The description included the impact of printing direction on the geometry of the obtained samples and tensile strength as well as the dependency of tensile strength on the sample thickness. The results can be useful for design engineers and process engineers designing thin-walled components produced with SLS. Thin samples were obtained with a considerable deviation spread of the actual dimension from the nominal one. It was found that the tensile strength of thin samples is much lower than those of standard cross-sections, which should be taken into account in the design of thin-walled elements.

## 1. Introduction

Rapid Prototyping methods are increasingly applied in various industries. They are an alternative manufacturing technology for complex, time-consuming and cost-intensive operations. These methods do not eliminate the traditional production methods, instead they perfectly support or complement them [1,2,3]. In recent years, the RPs have also become a subject of interest and research for many scientists. The reason for this is the wide range of 3D printing technologies and the extensive range of consumables materials. Complex three-dimensional components of any shape and application are produced during this process, designed in CAD programs and transferred to printing devices in a universal file format, e.g., *stl* [4,5]. In other words, this production method allows items to be manufactured without using a physical model. Currently, the most common 3D printing technology is Selective Laser Sintering (SLS) [6,7,8]. It uses various materials in powder form, e.g., polymers (polyamide, polystyrene) [9], metals (Al, Ti, Cu, Ni [10], alloys [11], ceramics (synthesis of aluminum oxide with zirconium [12], silicon-based ceramics [13]) and composite materials (Nylon-11 composites, polyethylene with very high molecular weight [14]). It consists of sintering the powdered material using an infra-red laser beam. When manufacturing a model from polymer powders, no additional support structures are needed. Powder from which the model is built, and which has not undergone the melting process, is the support material. At the end of the printing process, the temperature of the working chamber is reduced. After cleaning, the produced element is ready for use. Unused powder can be reused in subsequent manufacturing processes. The use of polymers as building blocks in 3D printing technology has many advantages, such as: high quality of manufactured products, great design freedom and high accuracy of manufactured elements, availability of materials which have found application and show great potential in the industry, printing complex elements or customization to the customer’s needs [15]. The mechanical properties and dimensional and shape accuracy of the final product depend on technological parameters, such as printing direction, laser speed and power, layer thickness, cooling time and temperature in the working chamber [16].

3D printing, also known as “additive manufacturing” (AM), “generative technology” (GT) is also used to produce small series of thin-walled machine and device parts on a micro to macro scale with high accuracy and precision [17,18,19,20]. This method works perfectly well when serious difficulties and limitations arise in a traditional technological process due to the time, resources and technical capabilities.

3D printing has become a popular manufacturing process of not only prototypes or models, but also components that are used in medical devices and pharmaceutical products [21]. Dental bridges are an example [22].

There are production technologies that enable manufacturing elements with geometrically complex internal structures, such as honeycomb structures, sponges or objects inside objects. One such technology is Fused Deposition Modelling (FDM) [23,24,25]. FDM is applied in medicine, for quick prototyping and reconstruction of, for example, the hip joint, based on magnetic resonance imaging. Based on a 3D model, stress and load simulations have been performed and an individual implant design has been developed [26].

3D printing is also used in microelectromechanical systems (MEMS) where components serve as sensors or actuators, e.g., in the latest mobile phones [27].

3D printing technology based on powder polymers has also some drawbacks and limitations. The manufactured elements are characterized by the anisotropy of the material, i.e., different mechanical properties in different directions, which affects their quality [28]. It is possible to optimize the settings of 3D printers to change the anisotropic properties of samples made [29]. The disadvantage of most 3D printing technologies are the relatively low mechanical properties compared to objects made in other technologies [30]. Therefore, it is worth combining printed elements with those made by other methods [31]. Components produced with SLS have a higher surface roughness than with alternative production methods such as casting, machining, injection molding etc. The processing conditions, the recovered powder used for production and the lower beam speed affect surface roughness [32].

As shown by the results of the research carried out earlier [33,34] by the authors on mechanical properties, models manufactured by selective laser sintering technology show clear differences in rheological properties such as stress relaxation during tensile and compression tests. In the work for the construction of the models, polyamide PA 2200 (based on PA 12) was used, the impact of the thickness of the constructed layer on the relaxation of compressive stresses, the quality of the surface layer and the weight of the models built were also described. It has been shown that increasing the thickness of the layer, while maintaining the same energy density transmitted to the sintered layer of the powder, reduces the mass of the produced models by over 20%. Obviously, this affects the lower strength properties, but in a predictable way that allows the prediction of these changes. Moreover, it has been shown that the samples manufactured with the sample layer thickness increased from 0.1 mm to 0.2 mm are much more susceptible to any other changes such as the printing direction or energy density. This is particularly evident in the case of pure PA 12 polyamide (PA 2200—EOS GmbH). Therefore, in the presented article, a material with glass fiber was used to build the sample models. Previous studies showed a clear influence of laser parameters on the mechanical properties and mass of the models produced, which is interesting because thanks to the possibility of a change in the thickness of the layer and the optimization of laser parameters, it is possible to produce models with increased layer thickness and reduced time while maintaining adequate strength. Moreover, in [35,36] it was shown that in laser additive technologies the printing direction affects both 2D and 3D roughness parameters but also a number of waviness parameters. Thus, it can be said that printing direction is a key technological parameter in almost all 3D/4D printing technologies.

As the engineering practice shows in the case of conventional manufacturing technologies such as casting, injection molding or machining, plastic-based materials show high isotropic of properties regardless of the casting direction or the type of machining. The method of turning, milling, injection molding does not significantly affect the strength of the models produced. In the case of 3D printing technology, the impact of the printing direction as shown by the current state of knowledge and the presented results is significant. The difference in mechanical properties results mainly from the nature of joining the layers of the material and not, as is the case in casting or injection molding, from its continuous casting and joining in the cooling process into one component. In the case of cutting, we do not damage the internal structure of the model, but only remove the material, which does not change its properties and does not affect its strength.

3D printers are used for commercial purposes in the production of functional components reinforced with carbon, glass or Kevlar fibers [37,38]. The use of composites reinforced with fibers or nanomaterials will allow the achievement of high performance and excellent functionality [9]. There is a continuous need for experimental research to determine the properties of fiber-reinforced components made in 3D technology. Therefore, it is important to conduct research aimed at determining the mechanical properties of such elements in terms of tension, compression and bending as well as fatigue and impact parameters [39]. PA 12 polyamide composite material, containing a low percentage of aluminum, is perfect for manufacturing medical devices, such as orthoses or biomodels [28,40]. The surface layer quality assessment and the dimension and shape comparative analysis of the finished prototype and intermediate elements obtained by 3D printing and the casting method are presented, for example, in the paper [41,42].

3D printing technologies, in particular selective laser sintering thanks to the use of many materials reinforced with, for example, glass fibers—PA 3200 GF, aluminum powder—Alumide, flame retardant additives—PrimePart FR, Polyamide 12 Filled with Glass Beads and Carbon Fibers—PA 640—GSL, PA 6, PA 11, Polyetherketoneketone—PEKK, Thermoplastic Elastomer—TPE, Polystyrene (PS) are widely used in Robotics. A perfect example of this are numerous prototype constructions in which thin-walled models allow the reduction of the weight of industrial robots, rovers or gripper elements, which are also used in medicine. The PA 3200 GF material, which was used to build test samples, is characterized by increased strength properties thanks to the use of glass fibers and, as the manufacturer announces, by much more isotropic mechanical properties. In the case of prototype thin-wall structures and the use of 3D printing technology, the optimization process becomes very complicated, and the optimization of the model in terms of strength and weight is difficult. The use of SLS technology and carbon fiber-reinforced polyamide can solve the above-mentioned problems, therefore the undertaken research topic seems to be justified.

In this article, thin polyamide composite samples with the addition of glass fiber (PA 3200 GF material) were tested. Samples were made with SLS in three printing directions X, Y, Z. The tensile strength for different thickness of the samples and the maximum strain were estimated on the basis of the tensile strength test. The calculations take into account the cross-section individually for each sample due to the considerable dimensional deviations of the obtained thin elements.

## 2. Materials and Methods

### 2.1. Method

Selective Laser Sintering—SLS (EOS) is one of the most common industrial 3D printing methods. In this technology, the model is manufactured layer by layer using a powder material with a grain diameter of several micrometers. Using CAD objects (then STL), the 3D model is divided into layers, where a laser beam is used in each layer to scan the geometry corresponding to the cross-section of the model. In addition, by scanning a given layer, the laser energy also penetrates into the previously created layer, joining them together. Then the built-up platform is lowered by the set layer thickness (0.1 mm) and another layer of built-up material in powder form is distributed. The entire process takes place in a chamber heated to 168–170 °C (for PA 3200 GF) and under nitrogen—5.5 bar.

### 2.2. Materials

The machine used to build the samples is a Formiga P100 from EOS (EOS GmbH, Krailling, Germany). The material used to build up the samples was polyamide PA 3200 GF (selected properties, see Table 1), which is based on a well-known construction material—polyamide PA 12. In addition, this powder, with a grain diameter of 60 μm, can be reused after mixing with fresh (unused powder). The material density before sintering, in powder form, was 0.61 g/cm^3^, density of laser-sintered part—1.23–1.28 g/cm^3^ [43]. The printing processing parameters were as follows: layer height *Lh*—0.1 mm, laser power *P*—21 W, laser scanning speed υ—2000 mm/s, hatch distance *h*—0.25 mm and beam diameter—0.42 mm. The following parameters using formula 1 generated an energy density *ED* of 0.07 J/mm^2^.

(1) $$ED=\frac{P}{\upsilon h}x,$$

where: *x* = *d/h*.

### 2.3. Preparation of SLS Samples

A solid model of the sample was drawn in 3D CAD software and saved in a digital file *.stl* using triangulation parameters in the export options: resolution—adjusted, deviation—tolerance 0.016 mm, angle—tolerance 1°. It is important to remember not to use too-low triangulation parameters as round shapes will not be obtained (in this case R radius of fillet) or those that are too-high due to the large files (.*stl*).

**Figure 1:**
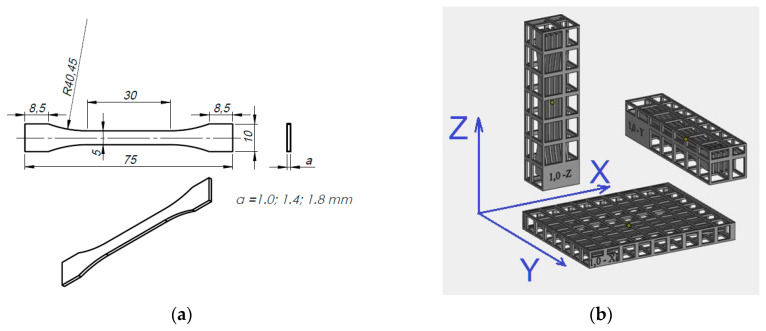

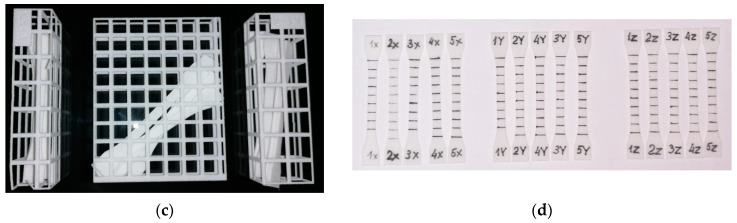
Samples built in three directions; (**a**) sample dimensions, (**b**) orientation on the build tray, (**c**) examples of printed samples in the cover baskets, (**d**) series of labelled samples prepared for the tensile strength test.

Figure 1 shows: a drawing of a single sample with its dimensions, a sample series orientations on the platform, made samples placed in working baskets and an exemplary series of samples made, marked and prepared to undergo a tensile strength test.

The thickness and width of each sample in the measuring base area was measured at three spots and the average thickness $\bar{a}$ and average width were calculated $\bar{b}$. Table 2 shows the measurement results of average thickness $\bar{a}$ and average width $\bar{b}$ of samples prepared for tensile test strength. The measurements were made with a micrometer with an accuracy of 0.01 mm. Sample identification includes: nominal thickness, i.e., the value designed and set for printing in the *.stl* file, the sample number in the series and the printing direction indication. For example, the identifier “1.8 2X” means a sample with a nominal thickness of *a* = 1.8 mm and the number “2” in the series, set in “X” direction on the working platform.

The measurements show that the average width and thickness values of the samples differ significantly from the nominal dimensions, i.e., dimensions designed when creating a 3D model of the samples.

### 2.4. Tensile Test

The tensile strength test was performed using a 3 kN Inspect mini (Hegewald & Peschke GmbH, Nossen, Germany) strength testing machine and an extensometer to measure strain. The test speed was set to 1 mm/min in the Labmaster software (Version 2.5.3.21) supplied with the Inspect mini.

The tensile strength *R_m_* was calculated by the machine software from the formula 2:

(2) $$R_{m}=\frac{F_{m}}{\bar{a}\bar{b}},$$

where: *F_m_*—maximum strength, $\bar{a}$—average measured sample thickness, $\bar{b}$—average measured sample width.

The average width and average thickness of the samples (see Table 2) were entered into Labmaster for each sample separately, so that the software can plot the tensile chart in the stress–strain coordinates and calculate *R_m_*. In these types of tests, the nominal values of the sample dimensions are usually entered in the programs for the entire series, while the values of the dimensional deviations are taken into account in the estimation of measurement errors. In the case of thin samples, such an approach would significantly distort the result of the entire sample.

The tensile modulus of elasticity was calculated with respect to the two values of the relative elongation (Equation (3)):

(3) $$E=\frac{\sigma_{2}-\sigma_{1}}{\varepsilon_{2}-\varepsilon_{1}},$$

where:

*E*—is the tensile modulus of elasticity, in MPa;
$\sigma_{1}$—is the stress, in MPa, measured at the value of the relative elongation $\varepsilon_{1}=0.0005$;
$\sigma_{2}$—is the stress, in MPa, measured at the value of the relative elongation $\varepsilon_{2}=0.0025$.

The standard deviation is calculated from the Formula (4):

(4) $$SD=\sqrt{\frac{1}{\left(n-1\right)}\sum_{i=1}^{n}{\left(x_{i}-\bar{x}\right)}^{2}},$$

where: *n*—sample size, $\bar{x}$—arithmetical mean of all measured values in a sample.

## 3. Results

**Figure 2:**
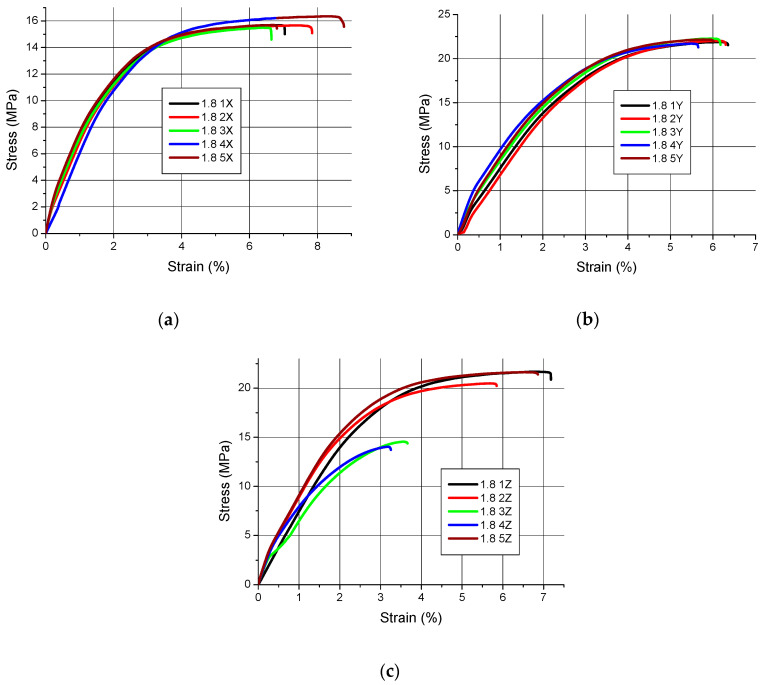
Tensile curves for samples with a nominal thickness of 1.8 mm (**a**) orientation X, (**b**) orientation Y, (**c**) orientation Z.

**Figure 3:**
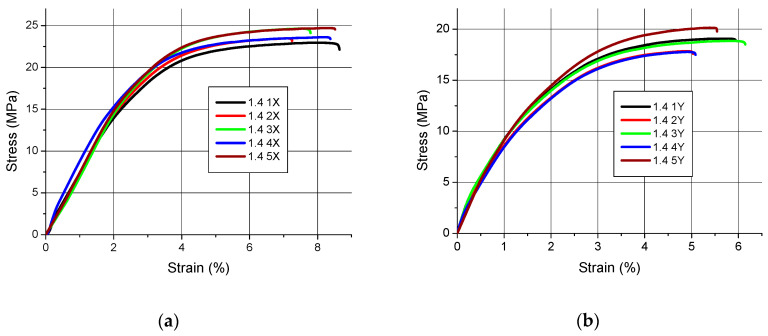

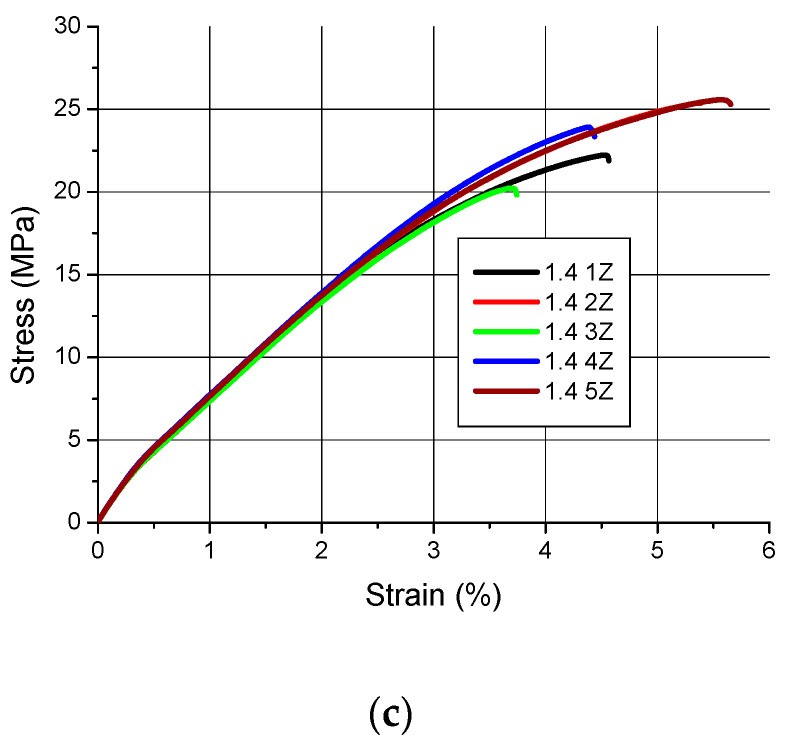
Tensile curves for samples with a nominal thickness of 1.4 mm (**a**) orientation X, (**b**) orientation Y, (**c**) orientation Z.

**Figure 4:**
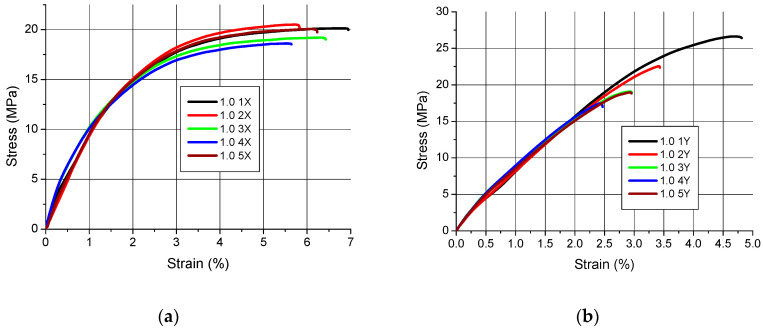
Tensile curves for samples with a nominal thickness of 1.0 mm (**a**) orientation X, (**b**) orientation Y, (**c**) orientation Z.

Tensile charts of samples in the stress–strain coordinate system are shown in Figure 2, Figure 3 and Figure 4.

The qualitative assessment of the tensile diagrams presented in Figure 2, Figure 3 and Figure 4 shows that slightly worse results were obtained for the samples manufactured in the Z orientation. This is clearly visible especially in Figure 2c and Figure 4c, where the curves do not all “**tear up**” and some samples undergo at fracture at a tension lower than expected. Moreover, the character of the tensile curves of all samples, regardless of their thickness, made in the X orientation is the same, i.e., the initial straight section (indicating a quasi-linear elasticity) followed by a curvature with final flattening and the end of the curves indicating a sample break. The only differences are in the value of the maximum breaking stress.

The tensile strength values *R_m_* and the maximum percentage deformation *ɛ_m_* that occurred during the maximum tensile force are shown in Table 3.

The values of modulus of elasticity for individual samples calculated on the basis of Equation (3) are presented in Table 4.

The highest mean value of the modulus of elasticity was obtained for samples with a thickness of 1 mm made in the Z orientation, and the smallest for samples with a thickness of 1.4 mm and the direction of printing X. The dispersion of results, which are characteristic for samples of small thicknesses, do not allow in this case the formulation of a clear thesis about the influence of the print direction on the obtained values of elasticity modulus.

**Figure 5:**
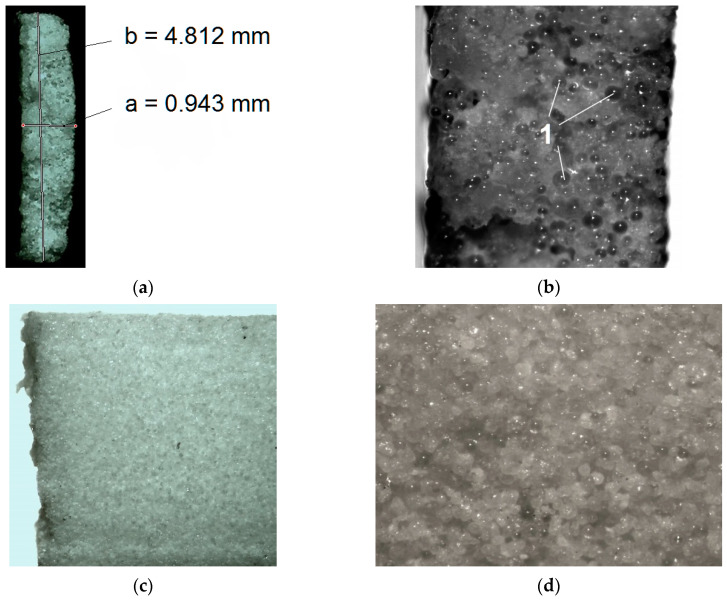
Photographs of the sample surface taken with a stereoscopic microscope; (**a**) cross-section of the broken sample with dimensions—6× magnification, (**b**) cross-section of the torn sample with visible glass powder grains (1)—50× magnification, (**c**) flat surface of the sample with the visible tear point—12× magnification, (**d**) flat surface of the sample—50× magnification.

Figure 5 shows an example of a cross-section of a broken sample at 6× magnification and macroscopic structures at different magnifications of a stereoscopic microscope.

The microscopic images at 50× magnification and the use of appropriate filters (Figure 5b,d) reveal the uneven distribution of glass microspheres, which is a component in the polymer base material. In the case of elements of small thickness, this type of structure may affect the dispersion of the obtained results of tensile strength and modulus of elasticity.

## 4. Discussion

When analyzing the obtained results, several important problems should be considered:

– Differences between the nominal (designed) dimensions of thin samples and the actual dimensions obtained by 3D printing (see Table 2), taking into account the orientation on the work platform;
– Impact of the orientation of elements on the working platform on the strength *R_m_* and the maximum percentage strain *ɛ_m_* (Table 3).

When evaluating the results of sample thickness and width measurements, it is best to use indicators of relative percentage differences of thickness Δ*a* and width Δ*b* for each measurement series. They are described by the Formulas (5) and (6):

(5) $$\Delta a_{X,Y,Z}=\frac{\left|a-{\bar{a}}_{X,Y,Z}\right|}{a}\cdot 100\%,$$

where: *a*—nominal sample thickness, e.g., *a* = 1.8 mm, ${\bar{a}}_{X,Y,Z}$—average sample thickness in a given measurement series based on Table 2, e.g., for the nominal thickness *a* = 1.8 mm and X orientation the value ${\bar{a}}_{X}$ = 1.82 mm.

(6) $$\Delta b_{X,Y,Z}=\frac{\left|b-{\bar{b}}_{X,Y,Z}\right|}{b}\cdot 100\%,$$

where: *b*—nominal sample width, e.g., *b* = 5 mm, ${\bar{b}}_{X,Y,Z}$—average sample width in a given measurement series based on Table 2, e.g., for the nominal width *b* = 5 mm and X orientation the value ${\bar{b}}_{X}$ = 4.93 mm.

**Figure 6:**
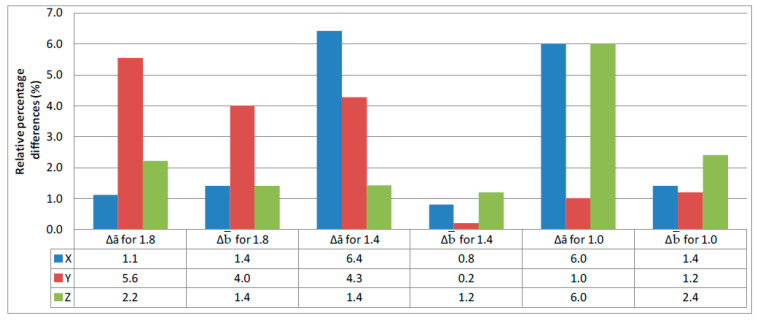
Relative percentage differences in sample thickness and width.

The calculated values of the relative percentage differences of thickness Δ*a* and width Δ*b* for individual measuring series calculated from the data in Table 2 are shown in Figure 6.

On the basis of Figure 6, it cannot be unequivocally stated that the differences between the nominal sample size and that obtained as a result of 3D printing depend on the orientation on the working platform of the machine. They were generally between 6.4% for the nominal sample thickness of 1.4 mm and the X orientation on the working platform and 1% for the nominal thickness of 1 mm and the Y orientation. Additionally, for the width, these differences were between 4% and 0.2% for the Y direction. The nominal width of all samples was the same and was 5 mm.

These dimensional differences are crucial for calculating the cross-sectional area of the samples and thus the stress, which is the ratio of the loading force to the value of the cross-sectional area. Therefore, when conducting tensile strength tests, individual thickness and width values should be entered for each sample individually.

In the case of tensile strength, it cannot be assumed that the orientation has a significant impact on its value. This is shown in Figure 2, Figure 3 and Figure 4 and the results in Table 3. However, as the charts in Figure 2c and Figure 4c show, the Z-orientation gives worse results because the three samples obtained such low tensile strength that it was treated as a gross error and these results were not included in the calculations. The above conclusions apply only to the thin-walled parts tested. As is known from other works [15,44,45,46] the orientation of the parts on the working platform has an impact on the tensile strength.

In this particular case, the tensile strength value may be significantly affected by the heterogeneity of the tested material, the component of which is glass powder. Macrostructures are visible in the photographs, as shown in Figure 5, and that the glass powder component is unevenly distributed in the base material space. Moreover, the adhesion of the polyamide powder to the grains of the glass powder is presumed to be adhesive. This weakens the strength of the obtained elements, especially in the case of thin-walled elements.

## 5. Conclusions

The tensile strength test results presented in this paper may be useful for design engineers designing thin-walled elements made with SLS. In the case of thin-walled elements, lower tensile strength values or higher values of the safety factor should be used in the calculation. Moreover, the values of elasticity coefficients calculated on the basis of the obtained tensile test results for thin samples are lower than those provided by the manufacturer. These results are likely to be influenced by the substantially thin thickness of the samples, which may mean that separate strength tests should be performed for thin components and included in international standards.

Furthermore, when designing the geometry, especially of thin-walled elements, one should take into account the differences between the nominal dimension and the actual dimension obtained as a result of the 3D manufacturing process. This allows the making of some dimensional adjustments at the design stage, such as using the data in Table 2. Of course, this only applies to the material used in this work for testing.

There was also a large result spread of the percentage elongation, from a minimum of 3.3% for 1-mm-thick Y-orientation samples to 8.1% for 1.4-mm-thick X-orientation samples. However, if the values of the standard strain are compared, it is generally the highest for the results obtained for 1-mm-thick samples, i.e., the thinnest.

## Figures and Tables

**Table 1:** Mechanical Properties of PA 3200 GF material.

Mechanical Properties	Value	Unit	Test Standard
Tensile Modulus	3200	MPa	ISO 527
Tensile Strength	51	MPa	ISO 527
Strain at break	9	%	ISO 527
Charpy impact strength (+23 °C)	35	kJ/m^2^	ISO 179/1eU
Charpy notched impact strength (+23 °C)	5.4	kJ/m^2^	ISO 179/1eA
Flexural modulus (23 °C)	2900	MPa	ISO 178
Flexural Strength	73	MPa	ISO 178
Izod notched impact strength (+23 °C)	4.2	kJ/m^2^	ISO 180/1A
Izod impact strength (+23 °C)	21	kJ/m^2^	ISO 180/1U
Shore D hardness	80	-	ISO 7619-1
Ball indentation hardness	98	MPa	ISO 2039-1

**Table 2:** Dimensions of the printed samples before tensile strength test.

Designation of the Samples	$\bar{a}$ (mm)	$\bar{b}$ (mm)	Designation of the Samples	$\bar{a}$ (mm)	$\bar{b}$ (mm)	Designation of the Samples	$\bar{a}$ (mm)	$\bar{b}$ (mm)
1.8 1X	1.83	4.94	1.4 1X	1.31	4.96	1.0 1X	1.05	4.93
1.8 2X	1.82	4.93	1.4 2X	1.31	4.97	1.0 2X	1.06	4.92
1.8 3X	1.82	4.93	1.4 3X	1.31	4.95	1.0 3X	1.07	4.93
1.8 4X	1.82	4.93	1.4 4X	1.31	4.95	1.0 4X	1.06	4.93
1.8 5X	1.82	4.93	1.4 5X	1.31	4.95	1.0 5X	1.06	4.94
$\bar{x}$	**1.82**	**4.93**	$\bar{x}$	**1.31**	**4.96**	$\bar{x}$	**1.06**	**4.93**
*SD*	**0.0045**	**0.0045**	*SD*	**0**	**0.0089**	*SD*	**0.0071**	**0.0071**
1.4 1Y	1.67	4.8	1.4 1Y	1.35	4.99	1.0 1Y	0.99	4.93
1.4 2Y	1.71	4.8	1.4 2Y	1.34	5	1.0 2Y	1	4.95
1.4 3Y	1.73	4.79	1.4 3Y	1.34	4.99	1.0 3Y	0.98	4.92
1.4 4Y	1.72	4.8	1.4 4Y	1.32	4.97	1.0 4Y	0.98	4.95
1.4 5Y	1.69	4.8	1.4 5Y	1.34	4.98	1.0 5Y	0.98	4.93
$\bar{x}$	**1.70**	**4.80**	$\bar{x}$	**1.34**	**4.99**	$\bar{x}$	**0.99**	**4.94**
SD	**0.0241**	**0.0045**	SD	**0.011**	**0.0114**	SD	**0.0089**	**0.0134**
1 1Z	1.77	4.98	1.4 1Z	1.36	4.93	1.0 1Z	0.95	4.86
1 2Z	1.79	4.95	1.4 2Z	1.39	4.95	1.0 2Z	0.95	4.88
1 3Z	1.75	4.87	1.4 3Z	1.38	4.94	1.0 3Z	0.94	4.89
1 4Z	1.74	4.89	1.4 4Z	1.37	4.94	1.0 4Z	0.93	4.89
1 5Z	1.75	4.95	1.4 5Z	1.40	4.95	1.0 5Z	0.95	4.88
$\bar{x}$	**1.76**	**4.93**	$\bar{x}$	**1.38**	**4.94**	$\bar{x}$	**0.94**	**4.88**
SD	**0.02**	**0.0460**	SD	**0.0158**	**0.0084**	SD	**0.01**	**0.0123**

**Table 3:** Tensile strength and maximum percentage strain.

Designation of the Samples	*R_m_* (MPa)	*ɛ_m_* (%)	Designation of the Samples	*R_m_* (MPa)	*ɛ_m_* (%)	Designation of the Samples	*R_m_* (MPa)	*ɛ_m_* (%)
1.8 1X	15.69	6.8	1.4 1X	22.96	8.6	1.0 1X	20.14	6.9
1.8 2X	15.67	8.8	1.4 2X	23.48	7.2	1.0 2X	20.51	5.8
1.8 3X	15.49	6.6	1.4 3X	24.65	7.8	1.0 3X	19.19	6.4
1.8 4X	16.35	7.8	1.4 4X	23.62	8.4	1.0 4X	18.61	5.6
1.8 5X	15.70	7.0	1.4 5X	24.71	8.5	1.0 5X	20.06	6.2
$\bar{x}$	**15.78**	**7.4**	$\bar{x}$	**23.88**	**8.1**	$\bar{x}$	**19.7**	**6.2**
SD	**0.33**	**0.91**	SD	**1.53**	**1.2**	SD	**1.56**	**1.0**
1.8 1Y	21.89	6.0	1.4 1Y	19.07	5.9	1.0 1Y	26.62	4.8
1.8 2Y	22.02	5.7	1.4 2Y	17.85	5.0	1.0 2Y	22.55	3.4
1.8 3Y	22.27	6.2	1.4 3Y	18.86	6.1	1.0 3Y	19.08	3.0
1.8 4Y	21.70	6.3	1.4 4Y	17.77	5.1	1.0 4Y	17.46	2.5
1.8 5Y	22.17	6.4	1.4 5Y	20.14	5.5	1.0 5Y	18.95	3.0
$\bar{x}$	**22.01**	**6.1**	$\bar{x}$	**18.74**	**5.5**	$\bar{x}$	**20.93**	**3.3**
SD	**0.45**	**0.56**	SD	**1.95**	**0.96**	SD	**7.37**	**1.75**
1.8 1Z	21.67	7.2	1.4 1Z	22.23	4.6	1.0 1Z	17.28	4.6
1.8 2Z	20.49	5.8	1.4 2Z	25.38	5.4	1.0 2Z	18.12	6.6
1.8 3Z	14.54 *	3.7 *	1.4 3Z	20.23	3.7	1.0 3Z	16.87	6.1
1.8 4Z	14.03 *	3.3 *	1.4 4Z	23.91	4.4	1.0 4Z	14.05 *	5.0 *
1.8 5Z	21.63	6.9	1.4 5Z	25.58	5.7	1.0 5Z	17.88	6.6
$\bar{x}$	**21.26**	**6.6**	$\bar{x}$	**23.47**	**4.8**	$\bar{x}$	**17.54**	**6.0**
SD	**0.95**	**1.04**	SD	**4.51**	**0.36**	SD	**1.71**	**2.4**

* The values marked with an asterisk were not taken into account in further calculations due to the gross error.

**Table 4:** Elastic modulus values.

Designation of the Samples	*E* (MPa)	Designation of the Samples	*E* (MPa)	Designation of the Samples	*E* (MPa)
1.8 1X	1071	1.4 1X	895	1.0 1X	1272
1.8 2X	845	1.4 2X	677	1.0 2X	960
1.8 3X	968	1.4 3X	641	1.0 3X	1476
1.8 4X	528 *	1.4 4X	1193	1.0 4X	1481
1.8 5X	1070	1.4 5X	912	1.0 5X	1221
$\bar{x}$	**988**	$\bar{x}$	**864**	$\bar{x}$	**1282**
SD	107	SD	221	SD	214
1.8 1Y	1027	1.4 1Y	1304	1.0 1Y	1082
1.8 2Y	529 *	1.4 2Y	1230	1.0 2Y	977
1.8 3Y	1126	1.4 3Y	1318	1.0 3Y	1102
1.8 4Y	1390	1.4 4Y	1059	1.0 4Y	1071
1.8 5Y	1133	1.4 5Y	1049	1.0 5Y	1067
$\bar{x}$	**1169**	$\bar{x}$	**1192**	$\bar{x}$	**1059**
SD	155	SD	130	SD	48
1.8 1Z	759 *	1.4 1Z	973	1.0 1Z	1530
1.8 2Z	1274	1.4 2Z	950	1.0 2Z	1365
1.8 3Z	1003	1.4 3Z	946	1.0 3Z	1485
1.8 4Z	1139	1.4 4Z	996	1.0 4Z	1274
1.8 5Z	1249	1.4 5Z	971	1.0 5Z	1403
$\bar{x}$	**1166**	$\bar{x}$	**967**	$\bar{x}$	**1411**
SD	123	SD	20	SD	100

* The values marked with an asterisk were not taken into account in further calculations due to the gross error.
